# Voltage-Gated Na^+^ Channels in Alzheimer’s Disease: Physiological Roles and Therapeutic Potential

**DOI:** 10.3390/life13081655

**Published:** 2023-07-29

**Authors:** Timothy J. Baumgartner, Zahra Haghighijoo, Nana A. Goode, Nolan M. Dvorak, Parsa Arman, Fernanda Laezza

**Affiliations:** Department of Pharmacology & Toxicology, The University of Texas Medical Branch, Galveston, TX 77555, USA; tjbaumga@utmb.edu (T.J.B.); zahaghig@utmb.edu (Z.H.); nagoode@utmb.edu (N.A.G.); nmdvorak@utmb.edu (N.M.D.); paarman@utmb.edu (P.A.)

**Keywords:** voltage-gated sodium channels, Alzheimer’s disease, excitability, hippocampus, neurodegeneration, plasticity, pharmacology

## Abstract

Alzheimer’s disease (AD) is the most common cause of dementia and is classically characterized by two major histopathological abnormalities: extracellular plaques composed of amyloid beta (Aβ) and intracellular hyperphosphorylated tau. Due to the progressive nature of the disease, it is of the utmost importance to develop disease-modifying therapeutics that tackle AD pathology in its early stages. Attenuation of hippocampal hyperactivity, one of the earliest neuronal abnormalities observed in AD brains, has emerged as a promising strategy to ameliorate cognitive deficits and abate the spread of neurotoxic species. This aberrant hyperactivity has been attributed in part to the dysfunction of voltage-gated Na^+^ (Nav) channels, which are central mediators of neuronal excitability. Therefore, targeting Nav channels is a promising strategy for developing disease-modifying therapeutics that can correct aberrant neuronal phenotypes in early-stage AD. This review will explore the role of Nav channels in neuronal function, their connections to AD pathology, and their potential as therapeutic targets.

## 1. Introduction

Alzheimer’s disease (AD) is a progressive neurodegenerative disorder classically characterized by the accumulation of amyloid beta (Aβ) plaques and hyperphosphorylated tau aggregates that disrupt synaptic function, ultimately culminating in synaptic decline and neurodegeneration [1]. Current FDA-approved small-molecule therapeutics for AD include acetylcholinesterase inhibitors [2] and NMDA receptor antagonists [3], which are effective in providing symptomatic relief but lack disease-modifying properties. FDA-approved monoclonal antibodies, such as aducanumab [4] and lecanemab [5], show efficacy in the clearance of Aβ, but there is a lack of evidence that they convincingly slow AD progression among large clinical populations. Thus, there remains an unmet need for the development of disease-modifying therapeutics for AD.

Accumulation of neurotoxic proteins in key brain regions induces neuronal deficits that are widely thought to be the cause of AD symptoms. The precise mechanisms of Aβ- and tau-mediated AD pathology remain to be elucidated, an issue which is further complicated by interpatient variability [6]. Nonetheless, AD is defined by the accumulation of Aβ and tau deposits [7]. Aβ deposition begins in the frontomedial and temporobasal areas, spreading then to the remaining neocortical regions [8]. Tau accumulation is first observed in the entorhinal cortex [9] and spreads successively into the hippocampus [10]. While intricacies of the relationship between Aβ and tau seeding and accumulation remain elusive, several studies suggest that Aβ may facilitate the seeding of tau [11,12,13,14].

The Aβ and tau proteins progressively accumulate at synapses, interrupting synaptic communication through the degeneration of dendritic spines [15], leading to axonal degeneration and eventual neuronal loss [16]. These phenomena progressively hinder the function of the hippocampal circuit, inducing deficits in long-term potentiation (LTP) and long-term depression (LTD), two forms of synaptic plasticity widely thought to be the basis of progressive memory loss in AD [17].

## 2. Hippocampal Hyperactivity in Early-Stage AD

Prior to global neurodegeneration and resultant progressive loss of memory associated with late stages of AD, hippocampal hyperactivity is observed in rodent models [18,19,20] as well as human patients [21,22,23,24]. Functional MRI studies indicate that patients with mild cognitive impairment (MCI) display increased hippocampal activation during memory-related tasks compared to healthy adults [25,26], and this phenomenon has emerged as a potential biomarker of mild cognitive impairment and early-stage AD [27,28].

This hyperactivity occurs prior to amyloid plaque deposition [20,29,30], positioning the phenotype as one of the first neurophysiological alterations in the AD brain. While there remain many questions to be answered regarding the precise mechanisms, origins, and consequences of this phenotype, it has emerged as a common feature in AD that precedes greater cognitive decline [31]. In support of this aberrant elevated neuronal activity as a precursor to AD, it has been has been linked to cognitive dysfunction and decreased memory performance [27,31,32] as well as the production and accumulation of Aβ and tau [27,33,34,35,36,37,38]. Moreover, amelioration of hippocampal hyperactivity using anti-epileptics, such as levetiracetam, has been shown to improve cognition and memory performance in rodent models and patients with MCI or early-stage AD [32,39,40]. Therefore, given its acute and longitudinal impacts on AD pathophysiology, correcting hippocampal hyperactivity represents a promising and potentially disease-modifying approach for AD treatment.

As described above, the hyperactivity phenotype is linked to various neuronal processes that accelerate the rate of AD progression. Therefore, evaluation of molecular contributors to the phenotype is warranted. On account of their centrality in initiating and propagating the action potential (AP) [41,42], in this review, we discuss the contribution of voltage-gated sodium (Nav) channels to the hyperactivity phenotype observed in early-stage AD, their function as the disease progresses, and their viability as therapeutic targets for the disease.

## 3. Overview of Nav Channels in AD Pathology

Broadly speaking, proper hippocampal function is mediated by the activity of glutamatergic principal neurons and GABAergic interneurons [43,44,45]. Thus, in order to ameliorate aberrant hyperactivity, the functional contributions of each neuronal subtype must be considered. Glutamatergic principal cells comprise the vast majority of hippocampal neurons and are densely packed into layers [46], notably the dentate gyrus (DG), CA3, and CA1 regions whose excitatory connections compose the trisynaptic circuit [47,48]. GABAergic inhibitory interneurons, despite only comprising ~10% of hippocampal cells [43,46], dramatically influence the activity of the hippocampal circuit [49]. Unlike principal cells, interneuron subtypes display significant intrinsic and morphological diversity and are disseminated throughout all hippocampal subfields [43]. The diversity of interneuron axonal projections lends them the capacity to synapse onto single cells or neuronal clusters throughout the hippocampal formation, providing essential GABAergic tone [49,50] and preventing excessive excitation of principal neurons through feedforward and feedback inhibition [43,50]. Hippocampal network dynamics rely on the balance between the excitatory and inhibitory signals (E/I balance) [51,52] produced by the aforementioned cell types. Therefore, pathological deviations in hippocampal activity can be ascribed to altered excitability of either principal cells or interneurons.

Nav channels, which are molecular determinants of neuronal excitability, consist of nine distinct isoforms (Nav1.1–Nav1.9) [53] that have varying expression profiles among different cell types [54,55,56]. Nav1.1 and Nav1.6 are of particular interest as they display distinctive enriched expression in hippocampal interneurons [57,58] and principal neurons [54,59], respectively, enabling the initiation and propagation of action potentials in these cell types. In early-stage AD, Nav1.1 and Nav1.6 display unique expression profiles and functional activity [29,60], contributing centrally to the aberrant excitability of interneurons and principal cells and resulting in hippocampal hyperactivity. Given the dichotomous functional roles of Nav1.1 and Nav1.6 channels in modulating hippocampal activity, the two isoforms will be considered separately. In this section, the physiological roles and disease-associated functions of Nav1.1 and Nav1.6 channels in their respective cell types will be discussed.

### 3.1. Nav1.1

GABAergic inhibitory interneurons comprise a small percentage of hippocampal neurons [46] but are able to contribute substantially to the regulation of hippocampal excitability due to their distribution and diverse axonal projections [43,44]. Hippocampal interneurons are classified into several major subtypes based on neuronal molecular expression, including somatostatin neurons, parvalbumin neurons, neuropeptide Y neurons, vasoactive intestinal peptide neurons, and cholecystokinin neurons [61]. Of these subtypes, somatostatin (SOM)- and parvalbumin (PV)-positive interneurons comprise a large majority (~70–80%) of the hippocampal interneuron population [62]. The Nav1.1 channel is predominantly expressed in inhibitory interneurons [60,63], where it serves as a key molecular determinant of intrinsic firing. With its subcellular localization in soma, axon initial segments (AIS), and axons, Nav1.1 channels facilitate the initiation and propagation of action potentials in these cells [63,64].

Loss-of-function mutations to Nav1.1 result in reduced Na^+^ current, causing reduced action potential firing in interneurons [57]. The imbalance caused by decreased interneuron activity can lead to seizures and is the leading cause of multiple epilepsies and other disorders, most notably Dravet’s syndrome [58,65]. It has been shown that epileptiform activity, particularly in the form of nonconvulsive seizures, is common in early-stage AD patients [66]. Multiple lines of evidence indicate that this epileptiform activity accelerates the rate of AD pathology and worsening of cognitive symptoms [66,67]. Along with displaying epileptiform activity, decreased levels of Nav1.1 expression have been observed across multiple transgenic AD mouse models [68,69,70] as well as AD patients [70], further supporting the pathophysiological role of this channel in AD. Early studies demonstrated that GABAergic interneurons are resistant to Aβ and tau protein deposition [71,72]. Highlighting this finding, in a study of the expression of hyperphosphorylated tau in AD patients, Blazquez-Llorca et al. found that of almost 4000 PV-positive interneurons in the hippocampal formation and entorhinal cortex analyzed, paired-helical filaments of tau were present in only two [73]. Despite GABAergic interneurons being resistant to Aβ and tau deposition [71,72], the activity of these neurons is dysfunctional in AD, and the precise pathophysiological mechanisms are the focus of many recent investigations [74,75,76,77].

One proposed mechanism by which GABAergic interneuron activity becomes compromised in AD is through the activity of β-site APP cleaving enzyme 1 (BACE1) [78], a secretase involved in Aβ production. Crucially, BACE1 activity and levels are significantly increased in the brains of AD patients, which is thought to contribute to the progression of AD by increasing Aβ [79,80,81]. In addition to its role in Aβ production, the Nav channel β2 subunit, which is covalently linked to the Nav1.1 alpha subunit, is a substrate for BACE1 [78]. Moreover, increased BACE1 causes a reduction in Nav1.1 cell surface expression [78], which would be expected to decrease the excitability of GABAergic interneurons. Consistent with such an expectation, PV interneurons in the hippocampus display reduced excitability in rodent models of AD [82]. Therefore, despite their apparent resistance to Aβ and tau pathology [71,72,73], depletion of Nav1.1 and suppression of its activity in hippocampal interneurons is potentially a major contributor to network dysfunction and cognitive deficits in AD [70].

### 3.2. Nav1.6

Contrarily to GABAergic interneurons, glutamatergic principal cells comprise a majority of neurons in the hippocampus [46]. Also, in contrast to interneurons, principal cells display a distinct layered organizational pattern with a relatively high level of anatomical uniformity [83,84]. Nav1.6 is extensively expressed in the adult human brain and is the predominant Nav isoform in hippocampal principal cells [85]. Nav1.6 displays a distinctive pattern of subcellular localization, with concentrated expression at the AIS and nodes of Ranvier in hippocampal principal neurons [59]. This pattern allows the channel to centrally regulate action potential initiation, propagation, and saltatory conduction in these cells [86] and govern the spike threshold of glutamatergic principal cells and contribute to synaptic integration [54]. This distinct role of Nav1.6 is due to its accumulation in the distal AIS and hyperpolarized voltage dependence of activation, setting it apart from other Nav channel isoforms at the AIS. Given the central role of Nav1.6 in regulating the action potential in excitatory neurons, both gain-of-function and loss-of-function mutations have been found to have pathogenic consequences [59]. Functional alterations to the Nav1.6 channel have been implicated in a number of neuropsychiatric disorders [59,87,88,89]. It has been observed that haploinsufficiency of the SCN8A gene results in cognitive impairment and has been linked to intellectual disability [90]. Conversely, gain-of-function mutations to Nav1.6, which promote aberrant high-frequency neuronal firing, are closely linked with epileptiform activity [88,91].

In the context of AD, Nav1.6 has emerged as the focus of many investigations regarding network abnormalities and disease progression [29,34,92,93]. It has been observed that acute exposure to soluble Aβ oligomers results in hyperactivity of CA1 pyramidal neurons [94]. Further, it was observed that soluble Aβ exposure selectively upregulated the expression and function of Nav1.6 [29] and that pyramidal cell hyperactivity was abolished following treatment with Nav channel blockers [94], implicating this Nav channel isoform as a driver of AD-related hyperactivity. Additionally, it is observed that amyloid precursor protein (APP) both directly interacts with Nav1.6 [93] and enhances Nav1.6 surface expression via a G protein-coupled JNK pathway [92]. Functionally, this results in the potentiation of Nav1.6-mediated Na^+^ currents [92]. These mechanisms provide convincing evidence for the contribution of Nav1.6 to the hyperexcitation of principal neurons in early AD and reveal novel, potentially synergistic pathways that drive AD pathology.

## 4. Therapeutic Potential of Nav Channels for AD

Nav1.1 and Nav1.6 channels have distinct roles in hippocampal hyperactivity and are potential therapeutic targets for early-stage AD [39,69]. However, current Nav channel modulators have the potential for off-target effects due to a lack of isoform selectivity. With the development of high-resolution Nav channel structures [72] and a better understanding of the biology of isoform-specific accessory proteins and signaling pathways [95], progress has been made towards developing targeted therapies that are selective for specific isoforms. This section will discuss Nav1.1 and Nav1.6 modulation mechanisms and potential therapeutic strategies for early AD.

### 4.1. Nav1.1 Activation

Increased BACE1 activity in early-stage AD reduces Nav1.1 cell surface expression, leading to decreased excitability of GABAergic interneurons in the hippocampus and contributing to increased excitatory/inhibitory tone. Restoring proper interneuron activity may help correct this imbalance. One potential therapeutic strategy for overcoming deficits in interneuron activity in the hippocampus is the transplantation of interneuron progenitor cells [96]. This involves the extraction of progenitor cells from the medial ganglionic eminence and transplantation into the brain of the affected individual [96]. Following the procedure, these cells integrate into the existing neuronal circuitry and mature into functional inhibitory interneurons. In AD rodent models, it has been observed that hippocampal transplantation of wild-type progenitor cells provides little to no improvement; however, transplantation of progenitor cells overexpressing the Nav1.1 channel results in improved behavior and cognitive performance [60]. Conversely, transplantation of Nav1.1-deficient progenitor cells impaired learning and behavioral functions [60]. Despite encouraging results following the transplantation of Nav1.1 interneuron progenitor cells in rodent models, the associated risks may hinder the translatability of this approach. Gene therapy represents an alternative strategy for the restoration of Nav1.1 activity and interneuron activity [97,98]. The feasibility of this approach has been demonstrated in rodent models, where voltage-gated potassium channels were delivered for the treatment of epilepsy or neuropathic pain [99,100]. Recent and promising developments involving engineered prokaryotic Nav channels for cardiac arrhythmias also illustrate the potential of this concept for clinical applications [101] and may inform future investigations regarding the delivery of neuronal Nav channels.

Cumulatively, these studies not only further indicate the importance of interneuron function during early AD but also establish that interneuron-based interventions for hippocampal hyperactivity are reliant on Nav1.1. Thus, pharmacologically potentiating Nav1.1 activity represents a promising therapeutic avenue for early-stage AD. There are several known classes of sodium channel activators, such as toxins from the venom of several organisms [102] or pyrethroid insecticides [103]. While therapeutic development from these agents is challenging due to their overall toxic effects, using these chemicals to selectively activate Nav channels allows for the demonstration of therapeutic benefit. For example, δ-theraphotoxin-Hm1a and δ-theraphotoxin-Hm1b (Hm1a and Hm1b) are toxins derived from the venom of the Heteroscodra maculata tarantula that potentiates the activity of the Nav1.1 channel selectively [104,105]. Hm1a-mediated activation of Nav1.1 is able to restore the function of inhibitory interneurons from Dravet syndrome mice without affecting the activity of excitatory neurons, and intracerebroventricular infusion of Hm1a in the Dravet syndrome rodent model rescued the animals from seizures and premature death [105]. While Hm1a and Hm1b exhibit high structural homology, further investigation revealed that Hm1b is more stable in biological fluids [106]. Electrophysiological studies revealed that the Hm1b peptide causes a hyperpolarizing shift in the voltage dependence of activation and delays fast inactivation of the Nav1.1 channel, increasing both peak and sustained Nav1.1 currents [106] through stabilizing interactions with its domain four voltage sensor [106]. While further optimization is required for clinical applications, these toxins demonstrate the value of Nav1.1 activation for restoring inhibitory interneuron activity in hyperexcitability disorders.

Given the considerable potential of Nav1.1 activators for Dravet syndrome and other excitability disorders, the development of small molecule Nav1.1 agonists has emerged as a rich area of investigation [102,107,108]. A 2019 high-throughput screening campaign conducted by Takeda Pharmaceutical Company led to the identification of a series of 4-phenyl-2-(pyrrolidinyl)-nicotinamide derivatives as potent and selective Nav1.1 activators [108], and structural optimization yielded a compound with favorable druglike properties that achieves brain concentrations comparable to its potency in vitro. While further investigations are required to determine the safety and translational of this compound’s effects, it holds potential as a therapeutic for epilepsy disorders and thus may be applied for hyperexcitability in early-stage AD.

### 4.2. Nav1.6 Inhibition

In contrast to Nav1.1, Nav1.6 is primarily expressed in excitatory neurons, and increased expression and activity of the channel are observed in AD brains as a result of Aβ oligomer exposure [29,92]. Knockdown of the Nav1.6 channel in AD rodents rescues LTP deficits and mitigates hippocampal hyperactivity in AD models, restoring memory-associated beta, gamma, delta, and theta waves to normal levels [34]. Additionally, knocking down Nav1.6 decreased both the number and size of Aβ plaques in these animals, providing evidence for the disease-modifying potential of Nav1.6 modulation [34]. The reduction in Aβ plaque accumulation is caused by reduced transcription of BACE1. When Aβ oligomers are present, genetic knockdown or pharmacological inhibition of Nav1.6 reduces BACE1 transcription and BACE1-mediated cleavage of APP, resulting in decreased Aβ production and plaque accumulation [34]. Intriguingly, while treatment with levetiracetam, an anti-epileptic with a mechanism independent of Nav channels, improves memory and cognitive performance, it fails to alter Aβ production or plaque accumulation [109]. Therefore, reducing hippocampal hyperactivity, specifically through modulation of Nav1.6, could provide both acute improvements to memory and cognition as well as simultaneously reducing AD pathophysiology.

NBI-921352 is a Nav channel inhibitor being investigated for the treatment of epilepsy caused by Nav1.6 gain-of-function mutations. It displays high selectivity for Nav1.6~130-fold greater potency for Nav1.6 over Nav1.1 and Nav1.2 isoforms [110]. By stabilizing Nav1.6 inactivated state, NBI-921352 inhibits persistent and resurgent currents, which are the source of hyperexcitability in pyramidal neurons [91]. Crucially, while NBI-921352 reduces excitability in principal cells, the activity of fast-spiking interneurons is spared [110]. Nav1.6 inhibition has shown promise in pre-clinical models of AD by correcting synaptic dysfunction, reducing Aβ plaque accumulation, and improving cognitive function [34]. Phase 1 trials indicate that NCI-921352 is well-tolerated in healthy adults and displays evidence of CNS activity [111]. Further studies are needed to assess NBI-921352’s efficacy in diseased individuals. However, increased expression and activity of Nav1.6 in early AD provides the opportunity for increased potency in diseased tissues, and functional studies illustrate the potential of Nav1.6 as a therapeutic target for the correction of aberrant hyperexcitability.

## 5. Alternative Strategies for Modulation of Nav Channels

**Figure 1:**
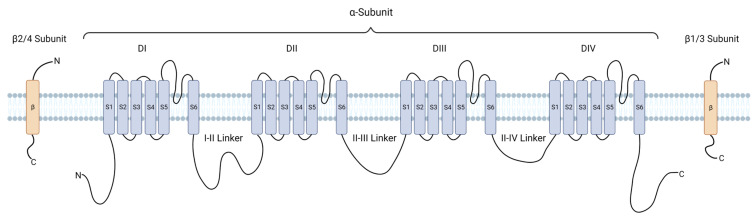
Schematic representation of Nav channel α- and β- subunit structure.

While the pore-forming α-subunit of Nav channels is primarily responsible for their physiological functions and has been the target for drug development of current Nav channel inhibitors, these channels rely on an array of intracellular channel-associated proteins (ChAPs) and post-translational modifications (PTMs) via various kinases for full function [112,113] (Figure 1, Table 1). These ChAPs have divergent regulatory effects and provide both isoforms as well as tissue specificity due to their unique intermolecular interactions with the channel as well their differential expression profiles in tissues [95,114,115,116]. In addition to selectivity, modulation of the Nav channel via ChAP complexes allows for bidirectional regulation of channel activity by either facilitating or inhibiting complex formation [115,117,118], therefore offering the potential for precise tuning of Nav channel activity through mechanisms beyond traditional agonism and inhibition. Promising ChAP modulators targeting specific regions, such as the C-terminal domain [113,114,119] of Nav channels, have been identified. These ChAP modulators hold the potential to pharmacologically modulate Nav channels with better precision and specificity. Additionally, many kinases have been intimately linked with intracellular ChAPs and exhibit altered functions or expression patterns in AD. Thus, targeting the functional interactions between the Nav channels, their interactors, and disease-associated kinase signaling pathways represents another novel strategy for AD drug development.

### 5.1. Modulation of Nav Channels via Kinase Signaling Pathways

In the AD brain, several kinase pathways are dysregulated, and the resultant alterations in signaling via these pathways are thought to contribute directly to AD progression [89,90,91]. Intriguingly, many of the kinases and associated signaling proteins linked with AD pathology are regulators of Nav1.1 or Nav1.6 and exert their functional effects via post-translational modifications (PTMs) and/or direct binding to the channel (Table 1) [92]. Thus, targeting the functional interactions between Nav1.1 or 1.6 and disease-associated proteins represents a novel strategy for AD drug development—and could allow for improved tissue selectivity over agents that modulate Nav channels alone. This section will explore several proteins with pathological links to AD, their regulatory effects on Nav channels, and how their interactions may be modulated to ameliorate hippocampal hyperactivity.

#### 5.1.1. Ca^2+^/Calmodulin-Dependent Protein Kinase

Calcium dysregulation during AD has long been investigated for its role in facilitating various facets of synaptic dysfunction and AD neuropathology [124,125,126,127]. Critically, aberrant alterations in Ca^2+^ signaling are detected in AD rodent models prior to severe memory impairments or histopathological biomarkers [124,128], indicating that correcting aberrant Ca^2+^ signaling and its downstream consequences may have disease-modifying potential. Increased Ca^2+^ levels in AD neurons occur through entry from extracellular space via Ca^2+^ permeable channels such as NMDA receptors [129] and voltage-gated calcium channels [130] or release from intracellular Ca^2+^ stores. These fluctuations in intracellular calcium are processed through the binding of Ca^2+^ ions to proteins such as calmodulin (CaM) [131]. The Ca^2+^/CaM-dependent protein kinases (CaMKs) are the primary binding partners of Ca^2+^/CaM complexes and are thus major effectors of alterations to Ca^2+^ ion flux in AD [132]. CaMKII, a member of the CaMK family, is a serine-threonine kinase that has altered expression and activity in the AD brain [133]. Following activation by Ca^2+^/CaM, CaMKII temporarily retains its kinase activity through autophosphorylation of its Thr286 residue [134]. The coupling of CaMKII with Nav channels has been shown to modulate excitability through the regulation of sodium currents across multiple Nav isoforms and cell types [135,136]. Notably, recent reports indicate that CaMKII phosphorylates Nav1.6, enhancing channel activity and Nav1.6-mediated neuronal excitability [121]. Moreover, inhibition of CaMKII is observed to ameliorate the pathological consequences of Nav1.6 gain-of-function mutations, which result in hyperexcitability disorders [137]. Therefore, inhibiting CaMKII-mediated phosphorylation of the Nav1.6 channel in early AD may represent a novel strategy to ameliorate hippocampal hyperactivity.

#### 5.1.2. Mitogen-Activated Protein Kinase

Mitogen-activated protein kinases (MAPKs) are a family of protein kinases best known for their contribution to various diverse cellular processes, including proliferation, inflammation, and apoptosis [138,139]. The MAPK family is comprised of 3 classes in mammals, extracellular-signal-regulated kinases (ERKS), Jun-amino-terminal kinases (JNKs), and stress-activated protein kinases (p38) [140]. The p38 MAPKs consist of 4 distinct isoforms: p38α, p38β, p38γ, and p38δ. Of particular interest, p38α is highly expressed in the cortex and hippocampus [141] and has been implicated as a regulator of synaptic plasticity [141,142]. In AD, elevated activity of p38 MAPKs is observed in the hippocampus during early phases [143,144], where they contribute to excitotoxicity and tau phosphorylation [145]. However, isoform-selective functional studies have revealed that the p38α isoform is likely the primary driver of AD pathogenesis [146], while other isoforms serve lesser pathogenic roles or potentially exert neuroprotective effects [147]. Nonetheless, given the consensus that p38α is a driver of AD pathology, inhibitors of this isoform have been evaluated and show some promise in ameliorating tau hyperphosphorylation, synaptic decline, AD-associated neuroinflammation, and cognitive impairment [148,149]. p38α has been identified as a direct regulator of Nav1.6 through phosphorylation of a Pro-Gly-Ser^553^-Pro motif intracellular loop L1, which results in reduced Nav1.6-mediated currents [150]. However, this effect is dependent upon the Pro-Gly-Tyr^1945^ motif of the Nav1.6 C-terminal tail, which is responsible for the binding of Nedd4-2 ubiquitin ligase and subsequent internalization of the channel [122]. Curiously, however, in the absence of a functional Pro-Gly-Try^1945^ motif, it is observed that p38 phosphorylation of Nav 1.6 enhances peak current density [122], indicating that p38 phosphorylation of Nav1.6 may have opposing effects on Nav1.6-mediated neuronal excitability dependent on the function of Nedd4-2. Haploinsufficiency of Nedd4-2 is linked to increased seizure susceptibility [151], which is a hallmark symptom of prodromal AD [30,66,67]. Thus, it is possible that dysfunctional Nedd4-2 reduces Nav1.6 internalization and allows functional upregulation of the channel via p38α, ultimately resulting in aberrant hyperexcitability. The precise functional alterations to p38α and Nedd4-2 and their resultant effects with Nav1.6 during early-stage AD remain to be fully elucidated. Nonetheless, the powerful modulation of Nav1.6 induced by p38α through S^553^ phosphorylation warrants further investigation, and modulation of this system may allow for the regulation of hippocampal activity in early AD.

#### 5.1.3. PI3K/AKT/GSK3β

The phosphatidylinositol 3-kinase (PI3K)/protein kinase B (AKT) pathway contributes to a broad range of physiological processes in the brain, including cell proliferation, differentiation, autophagy, and intraneuronal trafficking [152,153,154]. PI3K is the most upstream effector of this signaling cascade and is canonically activated by receptor tyrosine kinases in response to extracellular stimuli, followed by recruitment of its catalytic p110 subunit, which enables PI3K-medicated AKT phosphorylation [154,155]. Activated AKT then exerts modulatory effects on a myriad of downstream signaling molecules via phosphorylation and complexation, including mTOR, GABA receptors, and eukaryotic translation initiation factor α (eIF2α) [156,157], which mechanistically link the PI3K/AKT signaling axis to the regulation of synaptic plasticity. In AD brains, dysfunctional PI3K/AKT signaling has been linked to various pathogenic processes [153], including Aβ and tau pathology [158], neuroinflammation, impaired glucose metabolism, and oxidative stress [159], and restoration of its function may have neuroprotective effects [153]. AKT has also been identified as a regulator of Nav channels 1.1 [120] and 1.6 [160]. In the case of Nav1.1, it is observed that AKT directly phosphorylates the channel, resulting in decreased channel activity [120]. However, in CA1 pyramidal cells, Akt inhibition with triciribine results in increased action potential firing, which is likely driven by Nav1.6 [160]. This may be a result of direct interactions between AKT and Nav1.6 or a downstream effect of decreased activation of glycogen synthase kinase 3β (GSK3β), which phosphorylates the Nav1.6 channel at its T1936 residue [115], potentiating its activity. Moreover, GSK3β displays dysregulated function and increased expression in the hippocampus of AD patients and rodent models [161,162,163,164]. Therefore, in CA1 pyramidal neurons, activation of AKT may exert neuroprotective effects by reducing GSK3b activity, thereby decreasing Nav1.6 activity. However, considering the dichotomous effects of AKT activation on Nav1.1 [120] and 1.6 [160], inhibition of the downstream interaction between Nav1.6 and GSK3β may serve as a more viable approach than functional modulation of AKT activity.

### 5.2. Modulation of Nav Channel Macromolecular Complexes

In addition to regulation via phosphorylation, the Nav channel function is also modulated via stable interactions with other ChAPs [113,114,118]. One subclass of these accessory proteins that have been investigated for their ability to regulate Nav channel activity is the intracellular fibroblast growth factors (iFGFs) [95,117,123,165]. These proteins are a subset of the FGF superfamily and include FGF11, FGF12, FGF13, and FGF14 [166]. The iFGFs are expressed in excitable cells, where they are able to modulate the activity of Nav channels through stable interactions with their intracellular C-terminal domains [166]. Intriguingly, interactions of the iFGFs with Nav channels diverge among Nav and iFGF isoforms, resulting in an array of functional outcomes [113]. Despite this complexity, the divergence in cell-type expression among Nav channel isoforms may allow for fine-tuning of channel properties, and thus neuronal excitability, via modulating iFGF/Nav interfaces in target tissues. Of particular interest, FGF14 has been identified as a risk factor for AD [167,168]. In hippocampal neurons, it is observed that FGF14 overexpression results in a significant increase in Nav1.6 current densities, whereas genetic knockdown of FGF14 results in opposing phenotypes and decreased excitability [169]. Moreover, studies from our lab have illustrated that the FGF14/Nav channel complex is a target of GSK3β [170] and that pharmacological inhibition of GSK3β induces dissociation of the FGF14/Nav complex in hippocampal neurons and modifies FGF14-mediated modulation of Nav channel activity [170]. Further investigation revealed that GSK3β phosphorylates FGF14 at S226 and that phosphorylation of this residue is increased in Tg2576 AD rodents [171]. Moreover, alanine mutation of the S226 residue results in decreased complex formation with Nav1.6 [171]. As described in the previous section, GSK3β is a serine-threonine kinase with dysregulated activity and expression in the hippocampus of AD brains [161,162,163,164]. Thus, hyperphosphorylation of FGF14 by GSK3β may lead to a significantly increased probability of FGF14/Nav1.6 complex formation in early AD, thereby potentiating Nav1.6 activity and aberrant neuronal hyperexcitability in CA1 neurons. Further investigation is required to determine the precise mechanisms of phosphorylation-driven regulation of the FGF14/Nav1.6 complex, but disruption of FGF14 binding to the channel directly in the hippocampus of AD brains may prevent its potentiation of Nav1.6-mediated neuronal excitability and aid in ameliorating hippocampal network hyperactivity.

## 6. Conclusions

There remains a major need for AD therapeutics with disease-modifying properties. Therefore, elucidating the neuronal mechanisms that underlie AD progression is a critical step in the development of novel interventions. Hippocampal hyperactivity is one of the first neuronal phenotypes observed in AD patients, and it is linked to the onset of memory deficits as well progression of AD pathophysiology. Correction of this hyperactivity, therefore, is an attractive disease-modifying therapeutic strategy and has emerged as the focus of many recent investigations. While the complete roles of Nav1.1 and Nav1.6 throughout AD progression remain to be elucidated, their contributions to early-stage hyperactivity are under investigation and may prove critical to the development of disease-modifying AD therapeutics. Given their central roles in governing the excitability of these neuronal subtypes, functional modulation of Nav1.1 and Nav1.6 represents a promising therapeutic strategy to regulate hippocampal activity in early-stage AD, and their contributions to early-stage hyperactivity may prove critical to the development of disease-modifying AD therapeutics. In addition to pursuing traditional pharmacological approaches, Nav channel activity may be regulated through modulation of post-translational modifications or stable interactions with auxiliary proteins that alter channel activity. In the AD brain, there are numerous kinase signaling cascades and disease-related proteins that exhibit distinct functions and expression patterns during disease progression, several of which have established functional effects on Nav channels. In many cases, the functional consequences of these interactions are divergent among Nav isoforms. Therefore, functionally modulating Nav channels through altering their regulatory PTMs or protein complexes may provide the opportunity for the development of isoform-specific Nav channel therapeutics with improved specificity for diseased tissues.

## Figures and Tables

**Table 1:** Summary of Nav1.1 and Nav1.6 post-translational modifications (PTMs) and regulatory interactions with AD-associated proteins.

**Nav1.1 ChAPs and PTMs**				
	**Type of Interaction**	**Key Residues of Nav Channel**	**Functional Outcome**	**Reference**
AKT1	Phosphorylation of I-II Linker	S573, S684, S685, S704	Decreased Nav1.1 Activity	[120]
BACE1	Cleavage of β2 Subunit	C-terminus of β2 Subunit	Decreased Nav1.1 activity and α-subunit surface expression	[78]
**Nav1.6 ChAPs and PTMs**				
	**Type of Interaction**	**Key Residues of Nav Channel**	**Functional Outcome**	**Reference**
CaMKII	Phosphorylation of I-II linker	S561, S641, T642	Increased Nav1.6 Activity	[121]
p38 MAPK	Phosphorylation of I-II linker	S553	Decreased Nav1.6 Activity	[122]
FGF14	Binding to C-Terminal Domain	D1833, S1838, H1843, D1846, I1886, T1887, R1892	Increased Nav1.6 Activity	[113,118,123]
GSK3β	Phosphorylation of C-Terminal Domain	T1936	Increased Nav1.6 Activity	[115]

## Data Availability

No new data was created or analyzed in this study. Data sharing is not applicable to this article.
